# A young adult with leptospirosis associated acute inflammatory demyelinating polyneuropathy

**DOI:** 10.4102/sajid.v38i1.569

**Published:** 2023-12-21

**Authors:** Herman Bagula, Ismail Banderker, Muhammed S. Moosa

**Affiliations:** 1 Department of Internal Medicine, Faculty of Health Sciences, University of Cape Town, Cape Town, South Africa

**Keywords:** AIDP, leptospirosis, Guillain-Barré syndrome, polyneuropathy, post infectious

## Abstract

Leptospirosis is a zoonotic disease that commonly affects the liver and kidney. It can rarely affect the neurological system with aseptic meningitis being the commonest neurological presentation. We present the case of a patient with leptospirosis complicated by acute inflammatory demyelinating polyneuropathy.

**Contribution:**

To our knowledge, this is the first reported case of acute inflammatory demyelinating polyneuropathy as a complication of leptospirosis in South Africa.

## Introduction

Leptospirosis is a spirochaetal infection that is transmitted to humans from the urine of infected animals by direct or indirect contact. It usually affects the kidney and liver and rarely affects the neurological system.^1^ In this case report, we describe a patient with leptospirosis complicated by acute inflammatory demyelinating polyneuropathy (AIDP).

## Patient presentation

A 41-year-old HIV-negative male presented to a regional hospital in Cape Town, South Africa with a 5-day history of non-bloody and non-mucoid diarrhoea. He was unemployed and lived in an informal dwelling without access to municipal water and sanitation. He denied any recent exposure to river and dam freshwater or floodwaters. He denied any recent travel history, headache, fever, vomiting, sore throat or respiratory symptoms. He also complained of lower limb weakness that initially affected his ankles and then progressed to involve his knees, to the point where he was unable to walk. He denied any symptoms of bladder or bowel incontinence or retention. He denied any significant past medical or surgical history, alcohol or drug abuse and was not taking medication prior to presentation.

On admission to hospital, his diarrhoea had resolved, and on examination, he had a blood pressure of 86/54 mmHg, pulse 101, temperature 36.5°C, oxygen saturation on room air of 94% and respiratory rate of 18. He was jaundiced, dehydrated and without rash or lymphadenopathy. On neurological examination, he was alert and orientated, his cranial nerves were normal and he had no neck stiffness or muscle wasting. He had decreased tone in both lower limbs with symmetrical weakness (Medical Research Council grade 2/5 power) extending from L1 to S1 myotomes. He had absent ankle and patellar reflexes bilaterally, as well as loss of sensation (light touch and pain) extending up to L1 dermatome in both lower limbs. His upper limb power and reflexes were normal, his anal sphincter tone was normal and he had no cerebellar signs. His cardiovascular, respiratory and abdominal examinations were unremarkable.

His chest X-ray, 12-lead electrocardiography and urine dipstick showed no abnormalities. His blood results showed bicytopaenia (anaemia and thrombocytopenia), acute kidney injury, conjugated hyperbilirubinaemia and a mild transaminitis (see Table 1). His international normalised ratio, vitamin B12 and thyroid functions were normal, and serological testing for viral hepatitis A, B and C was negative. His sputum geneXpert^®^ (Cepheid, CA, USA) was negative for *Mycobacterium tuberculosis*, and his blood cultures were negative. His cerebrospinal fluid showed an elevated protein of 1.31 g/L, glucose of 3.9 mmol/L, polymorphs 4/uL, lymphocytes 7/uL, erythrocytes 25/uL. The gram stain, bacterial culture, TB culture and TB geneXpert^®^ on his cerebrospinal fluid (CSF) were negative. His serum Leptospira Enzyme Linked Immunosorbant Assay (ELISA) IgM was positive (titre of 3.8). Contrast Tomography (CT) scans of his head, chest and abdomen were normal and an Magnetic Resonance Imaging (MRI) of his spine showed no signs of arachnoiditis or other spinal cord or nerve root pathology. Nerve conduction study findings were compatible with a sensorimotor polyradiculopathy possibly because of AIDP (Table 1 and Table 2).

**TABLE 1:** Renal function tests, liver function tests and full blood count in a patient with leptospirosis-associated acute inflammatory demyelinating polyneuropathy.

Blood test	D0	D1	Day3	Day5	Day7	Day9	Day12†	Day33
Urea (mmol/L)	20.8	35.0	44.7	40.1	24.4	12.2	9.6	-
Creatinine (µmol/L)	499	681	713	653	389	205	150	118
eGFR (mL/min/1.73 m^2^)‡	12	8	7	8	16	34	45	66
Albumin (g/L)	27	27	-	31	-	-	36	-
Total bilirubin (µmol/L)	417	625	782	823	212	115	91	39
ALT (U/L)	105	108	112	83	71	84	97	43
AST (U/L)	320	276	173	-	-	64	61	30
ALP (U/L)	73	82	113	146	179	141	144	93
GGT (U/L)	98	133	340	371	-	197	160	106
White cell count (× 10^9^/L)	8.5	-	-	21.9	-	-	5.1	-
Haemoglobin (g/dL)	10.2	-	-	10.2	-	-	8.2	-
Platelets (× 10^9^/L)	39	-	-	267	-	-	342	-
INR	1.11	1.34	1.57	-	-	1.03	-	-

*Source:* Index patient’s results retrieved from South Africa’s National Health Laboratory Services

ALP, alkaline phosphatase; ALT, alanine aminotransferase; eGFR, estimated glomerular filtration rate; GGT, gamma-glutamyl transferase; INR, international normalised ratio.

† , day of discharge from hospital.

‡ , calculated using MDRD formula.

**TABLE 2:** Motor nerve conduction study in a patient with leptospirosis and lower limb weakness.

Nerve/site	Latency (ms)	Amplitude	Distances (mm)	Interval (ms)	Conduction velocity (m/s)
**Right peroneal**					
Ankle	**7.35 ms**	7.15 mV	-	7.35 ms	-
Head of fibula	**15.8 ms**	7.03 mV	340 mm	8.45 ms	40.2 m/s
Popliteal	**17.6 ms**	6.69 mV	410 mm	10.25 ms	40.0 m/s
**Right tibial**					
Ankle	6.4 ms	7.58 mV	-	6.40 ms	-
Popliteal	**17.55 ms**	7.59 mV	400 mm	11.15 ms	**35.9 m/s**
**Left peroneal**					
Ankle	**7.55 ms**	8.76 mV	-	7.55 ms	-
Head of fibula	**15.5 ms**	4.78 mV	330 mm	7.95 ms	41.5 m/s
Popliteal	**17.75 ms**	4.78 mV	410 mm	10.20 ms	40.2 m/s
**Left tibial**					
Ankle	**7.05 ms**	7.57 mV	-	7.05 ms	-
Popliteal	**18.1 ms**	7.09 mV	410 mm	11.05 ms	**37.1 m/s**
**Right median**					
Wrist	4.1 ms	7.75 mV	-	4.10 ms	-
Elbow	**9.54 ms**	7.15 mV	290 mm	5.44 ms	53.3 m/s
**Right ulnar**					
Wrist	3.5 ms	11.60 mV	-	3.50 ms	-
Below elbow	**9.36 ms**	10.51 mV	250 mm	5.86 ms	42.7 m/s
Above elbow	**11.1 ms**	10.52 mV	360 mm	7.60 ms	47.4 m/s

*Source:* Index patients nerve conduction studies results obtained from tertiary hospital’s neurology department

Note: Bold figures represent abnormal values.

In summary, our patient was diagnosed with leptospirosis complicated by AIDP. He was treated with intravenous ceftriaxone, intravenous 0.9% saline at 84 mL/h and had regular monitoring of his full blood count, renal function and liver profile. He received daily physiotherapy, thromboprophylaxis and close monitoring for any indication for haemodialysis. He was ambulatory by day 2 in hospital and at discharge, approximately 2 weeks after admission, his renal function and liver profile had improved significantly. When seen at the hospital outpatient department 2 weeks later, his renal function and liver profile were almost normal, and he was ambulating independently.

**TABLE 3:** Sensory nerve conduction study in a patient with leptospirosis and lower limb weakness.

Nerve/site	Latency (ms)	Amplitude	Interval (ms)
**Right sural**			
Sural	5 ms	19.20 uV	5.00 ms
**Left sural**			
Sural	5.04 ms	10.00 uV	5.04 ms
**Right median**			
Wrist	**2.62 ms**	**65.80 uV**	2.62 ms
**Right ulnar**			
Wrist	**2.82 ms**	13.60 uV	2.82 ms

*Source:* Index patients nerve conduction studies results obtained from tertiary hospital’s neurology department

Note: Bold figures represent abnormal values.

## Discussion

Leptospirosis is a zoonosis caused by *leptospires* and has a broad spectrum of clinical manifestations ranging from asymptomatic infection to fatal disease. In South Africa, the seroprevalence of Leptospira was between 9% and 12.5% from 2009 to 2011.^2,3^ Coastal and temperate conditions with wet winters and warmer summers appear to favour transmission of pathogenic leptospira species *L. borgpetersenii* and *L. interrogans* associated with rats.

The spirochetes are transmitted after direct contact with urine, blood or tissue from infected rodents. After an incubation period of 1–2 weeks, leptospirosis manifests as a biphasic illness consisting of initial leptospiraemia followed by an immune phase.^1^ Most leptospirosis is diagnosed by serology in South Africa as capacity for culture and Polymerase Chain reaction (PCR) is limited. Despite its reported variable diagnostic accuracy, the ELISA IgM is the test currently used to diagnose leptospirosis at the National Health Laboratory Services and was used to confirm the diagnosis in our patient.^4^

Most experts believe that the neurological complications of leptospirosis are secondary to the inflammatory response to the infection (immune phase), rather than direct invasion of the organism into the nervous system.^5^ This may explain why neurological manifestations occur later in the course of the illness.^5,6^ The pathophysiology of AIDP is thought to be because of molecular mimicry where antibodies against the infectious agent cross-react with gangliosides at nerve membranes resulting in nerve damage.^7^ Neurological manifestations of leptospirosis can affect both the central and peripheral nervous system and can present as: aseptic meningitis (the commonest manifestation), encephalitis, intracranial bleeds, cerebellitis, movement disorders, myelitis, paraplegias including AIDP, neuralgias, mononeuritis, autonomic lability and polymyositis.^1^ Data on the neurological manifestations of leptospirosis are commonly derived from hospital registries and its incidence has been reported to be approximately five per 100 patients admitted. Management of AIDP includes supportive care, intravenous immunoglobulin and plasma exchange.^7^

Neuroleptospirosis has variable clinical outcomes. Gokalp et al. described a 66-year-old rice farmer hospitalised with leptospirosis and renal failure and was treated with antibiotics and haemodialysis. Five days later, he developed AIDP for which he received total plasma exchange. He subsequently had complete neurological recovery and normalisation of his liver profile and renal functions.^8^ Gamage and Fernando reported a case of a 21-year-old man who was diagnosed with leptospirosis complicated by AIDP, respiratory failure requiring ventilatory support, thrombotic thrombocytopenia purpura and optic neuritis. After treatment with ceftriaxone, prednisone, haemodialysis, immunoglobulins and plasmapheresis, the patient had incomplete recovery of his neurology and was permanently blind at the time of discharge from hospital.^9^

Our patient presented with hepatorenal syndrome that is typical of leptospirosis, but with a neurological presentation not commonly associated with the disease. The differential diagnosis for the lower motor neurone pattern of weakness observed in our patient included myeloradiculopathy and AIDP, the latter being confirmed with nerve conduction studies. Organisms commonly associated with AIDP include *Campylobacter jejuni*, Epstein-Barr virus (EBV), cytomegalovirus (CMV) and *Mycoplasma pneumonia*.^10^ We did not test for EBV or CMV because these pathogens are associated with malignancy, HIV infection and other immune compromised states in adults and our patient had none. We were unable to exclude *Campylobacter jejuni* on stool culture because our patient’s diarrhoea had resolved by the time of presentation. After confirmation of negative blood and CSF cultures, as well as review of all investigations, we made an overarching assessment that our patient’s neurological syndrome was likely because of his primary diagnosis of leptospirosis. He demonstrated rapid recovery of muscle strength, had no autonomic or respiratory dysfunction and therefore did not require treatment with intravenous immunoglobulin or plasmapheresis. By the time of discharge from hospital, his renal function had improved significantly without requiring haemodialysis, his liver profile was near to normal and he was independently ambulatory.

## Conclusion

Neurological manifestations of leptospirosis are uncommon but should be considered in anyone diagnosed with leptospirosis and presenting with focal neurological signs. Furthermore, because of its rarity, clinicians should carefully exclude alternative causes for neurological symptoms and signs in patients with leptospirosis. Lastly, patients should be closely monitored during hospital admission because complications arising from neuroleptospirosis can be catastrophic and permanent if not managed early.

## Interpretation of nerve conduction study results

Bilateral peroneal motor nerves have prolonged distal latencies, normal amplitudes and reduced conduction velocity.

Bilateral tibial motor nerves have prolonged distal latency, normal amplitudes and reduced conduction velocity.

Right median and ulnar motor nerves have normal distal latencies, amplitudes and conduction velocity.

Bilateral sural sensory responses have normal distal latencies and amplitudes.

Right median and ulnar sensory nerves have mildly prolonged distal latencies with normal amplitudes.

The nerve conduction studies are in keeping with a symmetrical lower limb sensorimotor polyradiculopathy, evidenced by globally prolonged distal latencies, reduced conduction velocities and absent F responses.
